# Supplementary material for: Fatty Acid-Binding Proteins Aggravate Cerebral Ischemia-Reperfusion Injury in Mice
Source: Biomedicines. 2021 May 10;9(5):529. doi: 10.3390/biomedicines9050529 (PMC8150391; doi:10.3390/biomedicines9050529)
Supplement: Supplementary file 1 [file biomedicines-09-00529-s001.zip › biomedicines-1171043-supplementary.pdf]

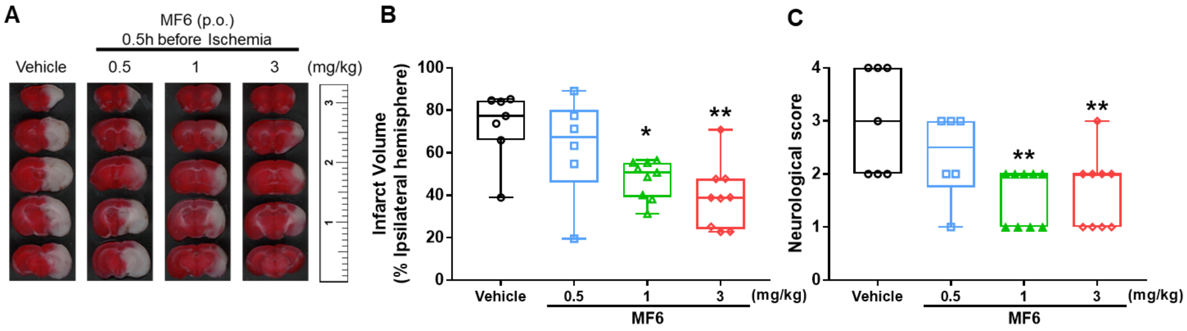

Figure. S1. Effects of MF6 pre-treatment on ischemia/reperfusion injury in mice. Mice were subjected to tMCAO for 2 h. MF6 was administrated with different concentrations (0.5, 1, 3 mg/kg) 30 min before reperfusion. Representative images of TTC staining (A), quantitative analysis of infarct volume (B) and neurological deficits (C) 24 h after reperfusion (n=6-9). Each data column represents the mean ± S.E.M. *p < 0.05, **p < 0.01 vs. I/R-treated with CMC group (Vehicle).

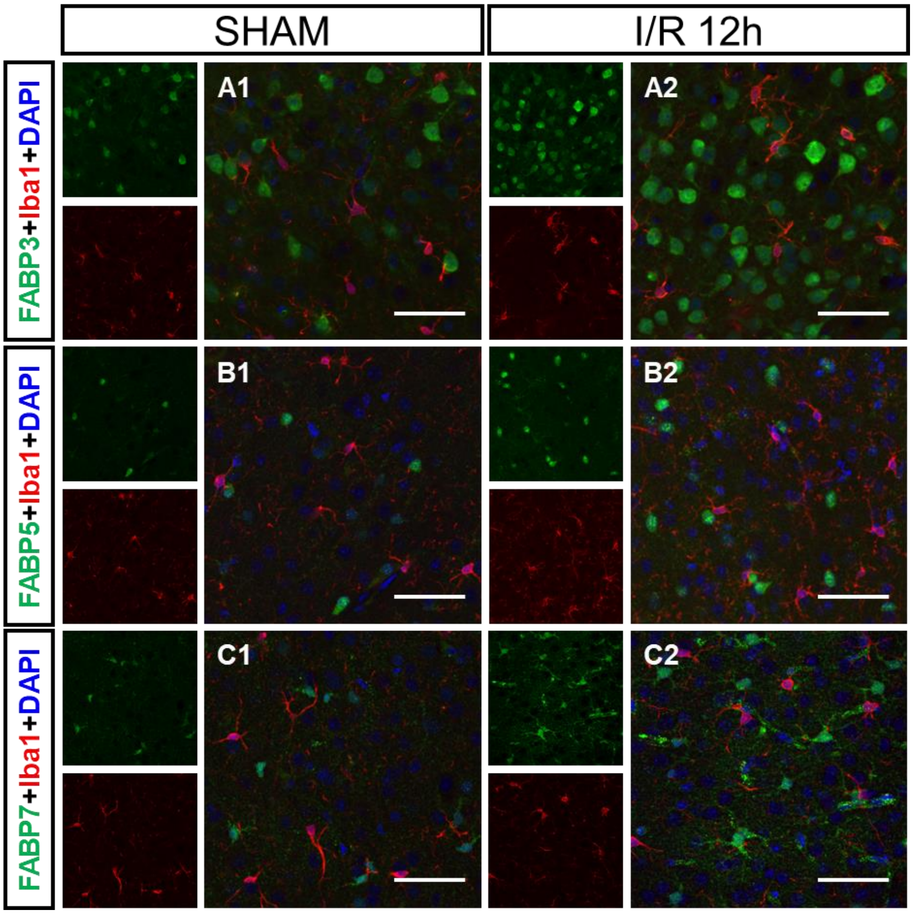

Figure. S2. Immunofluorescence of FABP3, FABP5 and FABP7 proteins, as well as Iba1 (a marker of microglia), in the cortexes of sham and ischemia/reperfusion mice. Representative micrographs of immunostaining FABP3 (green, A), FABP5 (green, B), and FABP7 (green, C) with Iba1 (red) in the cortical penumbra regions of sham mice and I/R mice (ipsilateral) at 12 h after reperfusion. Scale bar = 50 μm.

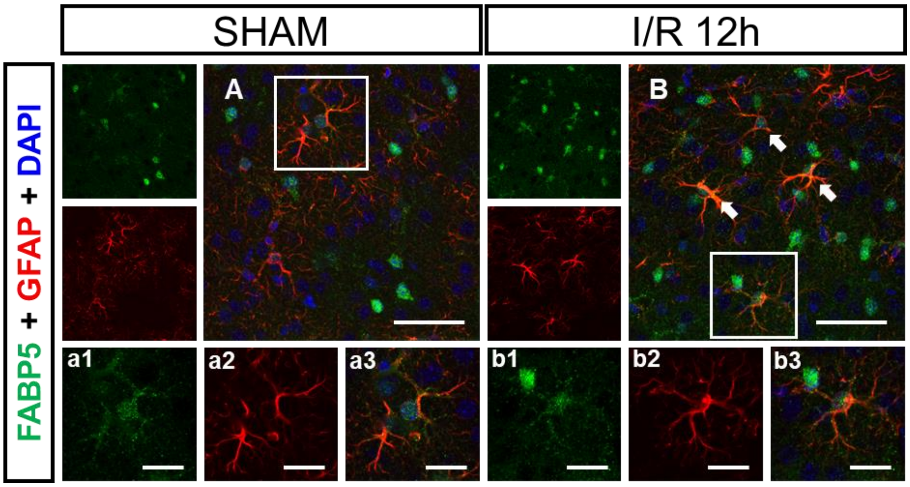

Figure. S3. Immunofluorescence of FABP5 with GFAP in the cortex of sham and ischemia/reperfusion mice. Representative micrographs of immunostaining with FABP5 (green) and GFAP (an astrocyte marker, red) in the cortical penumbra region at 12 h after reperfusion in sham mice (A) and I/R mice (ipsilateral) (B). Scale bar = 50 μm. Magnified images of the regions in the white boxes are shown in sub-panels a and b. Scale bar = 20 μm.
